# Supplementary material for: The Relation Between the Public Attitude Towards COVID-19 and its Applied Policies – a Dataset for Binational and Temporal Comparison
Source: J Open Psychol Data. 2023 Jul 14;11:12. doi: 10.5334/jopd.84 (PMC12269822; doi:10.5334/jopd.84)
Supplement: Appendix. — In the appendix demographic information about the participants’ place of residence in the two surveys is displayed. [file jopd-11-84-s1.pdf]

Appendix

Consent form study 1 German (original)

Liebe*r Teilnehmer*in,

aufgrund des Coronavirus (SARS-CoV-2) war das Jahr 2020 bisher in vielen Bereichen sehr außergewöhnlich. Wir führen diese Onlineumfrage durch, um mehr über die Auswirkungen der Pandemie auf Deutsche und Schweizer*innen in dieser speziellen Situation zu erfahren. Das Ziel dieser Studie ist es, einen Vergleich zwischen den Bürger*innen dieser zwei Länder in Bezug auf ihre Risikowahrnehmung und Einstellung zu den Corona-Massnahmen zu ziehen.

Die Teilnahme an der Studie ist freiwillig und das Ausfüllen des Fragebogens nimmt ungefähr 15 Minuten in Anspruch. Ihre Angaben bleiben anonym, werden vertraulich behandelt und lediglich für wissenschaftliche Zwecke ausgewertet. Es steht Ihnen jederzeit frei, die Umfrage abzubrechen, ohne dass für Sie dadurch Nachteile entstehen. Die Teilnahme an dieser Studie ist mit keinerlei Risiken verbunden.

Bitte lesen Sie sich die Fragen sorgfältig durch. Wenn Sie diese beantworten, denken Sie daran, dass es keine richtigen oder falschen Antworten gibt: Wir interessieren uns für Ihre Einschätzungen und Erfahrungen.

Teilnehmen kann jeder ab einem Alter von 18 Jahren mit einem festen Wohnsitz in Deutschland oder der Schweiz.

Am Ende des Fragebogens können Sie ihre E-Mail Adresse angeben, falls Sie über die Resultate der Studie informiert werden wollen, sobald diese ausgewertet worden sind.

Vielen Dank für Ihr Interesse und Ihre Teilnahme!

Consent form study 1 English

Dear Participant,

Due to the coronavirus (SARS-CoV-2), the year 2020 has been very exceptional in many areas so far. We are conducting this online survey to learn more about the impact of the pandemic on Germans and Swiss in this particular situation. The aim of this study is to draw a comparison between the citizens of these two countries in terms of their risk perception and attitude towards the Corona measures.

Participation in the study is voluntary and the questionnaire takes about 15 minutes to complete. Your information will be treated confidentially and analysed for scientific purposes only. You are free to cancel the survey at any time without any disadvantages for you. Participation in this study is not associated with any risks.

Please read the questions carefully. When you answer them, remember that there are no right or wrong answers: We are interested in your opinions and experiences.

Anyone over the age of 18 with a permanent place of residence in Germany or Switzerland can take part.

At the end of the questionnaire, you can enter your e-mail address if you would like to be informed about the results of the study as soon as they have been analysed.

Thank you for your interest and participation!

Consent form study 2 German (original)

Liebe*r Teilnehmer*in,

aufgrund des Coronavirus (SARS-CoV-2) war das letzte Jahr in vielen Bereichen sehr außergewöhnlich. Wir führen diese Onlineumfrage durch, um mehr über die Auswirkungen der Pandemie auf Deutsche und Schweizer*innen in dieser speziellen Situation zu erfahren. Das Ziel dieser Studie ist es, einen Vergleich zwischen den Bürger*innen dieser zwei Länder in Bezug auf ihre Risikowahrnehmung und Einstellung zu den Corona-Massnahmen zu ziehen. Es kann sein, dass Sie letzten Sommer diese Umfrage schon einmal ausgefüllt haben. Dies macht überhaupt nichts aus. Wir interessieren uns alleinfür ihre momentane Einstellung und Befindlichkeit.

Die Teilnahme an der Studie ist freiwillig und das Ausfüllen des Fragebogens nimmt ungefähr 5-10 Minuten in Anspruch. Ihre Angaben bleiben anonym, werden

vertraulich behandelt und lediglich für wissenschaftliche Zwecke ausgewertet. Es steht Ihnen jederzeit frei, die Umfrage abzubrechen, ohne dass für Sie dadurch Nachteile entstehen. Die Teilnahme an dieser Studie ist mit keinerlei Risiken verbunden.

Bitte lesen Sie sich die Fragen sorgfältig durch. Wenn Sie diese beantworten, denken Sie daran, dass es keine richtigen oder falschen Antworten gibt: Wir interessieren uns für Ihre Einschätzungen und Erfahrungen.

Teilnehmen kann jeder ab einem Alter von 18 Jahren mit einem festen Wohnsitz in Deutschland oder der Schweiz.

Vielen Dank für Ihr Interesse und Ihre Teilnahme!

Consent form study 2 English

Dear Participant,

Due to the coronavirus (SARS-CoV-2), the last year has been very exceptional in many areas. We are conducting this online survey to learn more about the impact of the pandemic on Germans and Swiss in this particular situation. The aim of this study is to draw a comparison between the citizens of these two countries in terms of their risk perception and attitude towards the Corona measures. You may have already filled in this survey last summer. This is not a problem.

We are only interested in your current attitude and state of mind.

Participation in the study is voluntary and the questionnaire takes about 15 minutes to complete. Your information will be treated confidentially and analysed for scientific purposes only. You are free to cancel the survey at any time without any disadvantages for you. Participation in this study is not associated with any risks.

Please read the questions carefully. When you answer them, remember that there are no right or wrong answers: We are interested in your opinions and experiences.

Anyone over the age of 18 with a permanent place of residence in Germany or Switzerland can take part.

Thank you for your interest and participation!

Overview Items

Table 4

Items of the First and the Second Survey

Topic	Survey 1 Items	Survey 2 Items

Seriousness Check	Möchten Sie ernsthaft in dieser Studie teilnehmen?	Möchten Sie ernsthaft in dieser Studie teilnehmen?
Demographic Questions	Geschlecht	Geschlecht
	Wie alt sind Sie?	Wie alt sind Sie?
	In welchem Land wohnen Sie?	In welchem Land wohnen Sie?
	In welchem Bundesland wohnen Sie? / In welchem Kanton wohnen Sie?	In welchem Bundesland wohnen Sie? / In welchem Kanton wohnen Sie?
Corona Status	-	Corona-Status
General Attitude	Ich fühle mich einer Risikogruppe zugehörig.	Ich fühle mich einer Risikogruppe zugehörig.
	Ich schätze meine persönliche, gesundheitliche Gefährdung durch das Coronavirus als niedrig ein.	-
	Ich halte es für sinnvoll mich an die vorgegebenen Regeln (z.B. Mindestabstand) zu halten.	Ich halte es für sinnvoll mich an die vorgegebenen Regeln (z.B. Mindestabstand) zu halten.
	Meine Besorgnis um Corona ist teilweise abhängig von der Strenge der offiziellen Maßnahmen.	Meine Besorgnis um Corona ist teilweise abhängig von der Strenge der offiziellen Maßnahmen.
	Ich habe Angst, dass jemand aus meiner Familie/meinem Freundeskreis aufgrund einer Infektion mit dem Coronavirus erkrankt.	Ich habe Angst, dass jemand aus meiner Familie/meinem Freundeskreis aufgrund einer Infektion mit dem Coronavirus erkrankt.
	Ich empfinde das Coronavirus *nicht* als gesundheitliche Bedrohung für mich und meine Familie/Freunde.	-
	Ich halte die Corona Maßnahmen für angemessen.	Ich halte die Corona Maßnahmen für angemessen.
	Ich finde die Corona Maßnahmen, die in meinem Land gelten, übertrieben.	Ich finde die Corona Maßnahmen, die in meinem Land gelten, übertrieben.
Mask-Wearing	Ich halte eine allgemeine Maskenpflicht in öffentlichen **Bereichen** für sinnvoll.	Ich halte eine allgemeine Maskenpflicht in öffentlichen **Innenräumen (Museen, Einkaufszentren, Restaurants, etc.)** für sinnvoll.
	Ich halte eine allgemeine Maskenpflicht in öffentlichen Verkehrmitteln für sinnvoll.	-

	**Ich halte eine allgemeine Maskenpflicht in Einkaufsläden (bspw. Supermärkten, Kleiderläden) für sinnvoll.**	**Ich halte es für sinnvoll in öffentlichen Aussenbereichen (Parks, Einkaufsgassen) eine Maske zu tragen.**
	Ich halte es für sinnvoll eine Maske **in öffentlichen Bereichen zu tragen** um mich selbst zu schützen.	Ich halte es für sinnvoll eine Maske zu tragen um mich selbst zu schützen.
	Ich halte es für sinnvoll eine **Maske in öffentlichen Bereichen zu tragen** um meine Mitmenschen zu schützen.	Ich halte es für sinnvoll eine Maske zu tragen um meine Mitmenschen zu schützen.
Private Meetings	Ich halte es für sinnvoll Treffen im öffentlichen Raum auf eine Anzahl an Personen zu beschränken.	-
	Ich besuche öffentliche Orte weniger oft als vor Covid-19, um das Risiko einer Ansteckung mit dem Coronavirus zu reduzieren.	-
	Ich bin der Meinung, dass eine Kontaktbeschränkung im öffentlichen Raum bei der Eindämmung des Virus hilfreich ist.	-
	Ich bin der Meinung, dass alle Kontaktbeschränkungen bei privaten Treffen im öffentlichen Raum aufgehoben werden sollten.	Ich bin der Meinung, dass alle Kontaktbeschränkungen bei privaten Treffen im öffentlichen Raum aufgehoben werden sollten.
Crowds	Ich halte es für sinnvoll die Personenanzahl bei Großveranstaltungen zu beschränken.	-
	Ich bin der Meinung, dass es aufgrund der Lage untersagt sein sollte in Clubs zu gehen.	-
	Ich bin der Meinung, dass die Regulierungen für Clubs aufgehoben sein sollten.	Ich bin der Meinung, dass die Regulierungen für Clubs aufgehoben sein sollten.
Communication	Ich nehme das Thema Coronavirus als präsent in den Medien wahr.	Ich nehme das Thema Coronavirus als präsent in den Medien wahr.
	Ich nehme das Thema Coronavirus als präsent in der Politik wahr.	Ich nehme das Thema Coronavirus als präsent in der Politik wahr.
	Ich nehme das Thema Coronavirus als präsent in Gesprächen mit	Ich nehme das Thema Coronavirus als präsent in Gesprächen mit

	meinem Mitmenschen wahr.	meinem Mitmenschen wahr.
	Ich halte mich für informiert bezüglich der derzeitig geltenden Maßnahmen.	Ich halte mich für informiert bezüglich der derzeitig geltenden Maßnahmen.
	Informationsquellen	Informationsquellen
Demographic Questions	Sind Sie Raucher*in?	Sind Sie Raucher*in?
	Leiden Sie an einer oder mehreren der folgenden Krankheiten oder haben Sie ein geschwächtes Immunsystem (wegen einer Therapie, Krankheit)?	Leiden Sie an einer oder mehreren der folgenden Krankheiten oder haben Sie ein geschwächtes Immunsystem (wegen einer Therapie, Krankheit)?
E-Mail	E-Mail	-

Note. All differences in item formulations between the first and the second survey are printed in bold.
